# Comparative genomics and host range analysis of four *Ralstonia pseudosolanacearum* strains isolated from sunflower reveals genomic and phenotypic differences

**DOI:** 10.1186/s12864-024-10087-7

**Published:** 2024-02-19

**Authors:** Shanwen Ding, Zijun Ma, Lin Yu, Guobing Lan, Yafei Tang, Zhenggang Li, Zifu He, Xiaoman She

**Affiliations:** Guangdong Provincial Key Laboratory of High Technology for Plant Protection, Key Laboratory of Green Prevention and Control on Fruits and Vegetables in South China Ministry of Agriculture and Rural Affairs, Plant Protection Research Institute, Guangdong Academy of Agricultural Sciences, Guangzhou, Guangdong 510640 China

**Keywords:** *Ralstonia pseudosolanacearum*, Sequevar, Genome, Genetic diversity, Sunflower

## Abstract

**Background:**

Bacterial wilt caused by *Ralstonia solanacearum* species complex (RSSC) is one of the devastating diseases in crop production, seriously reducing the yield of crops. *R. pseudosolanacearum,* is known for its broad infrasubspecific diversity and comprises 36 sequevars that are currently known. Previous studies found that *R. pseudosolanacearum* contained four sequevars (13, 14, 17 and 54) isolated from sunflowers sown in the same field.

**Results:**

Here, we provided the complete genomes and the results of genome comparison of the four sequevars strains (RS639, RS642, RS647, and RS650). Four strains showed different pathogenicities to the same cultivars and different host ranges. Their genome sizes were about 5.84 ~ 5.94 Mb, encoding 5002 ~ 5079 genes and the average G + C content of 66.85% ~ 67%. Among the coding genes, 146 ~ 159 specific gene families (contained 150 ~ 160 genes) were found in the chromosomes and 34 ~ 77 specific gene families (contained 34 ~ 78 genes) in the megaplasmids from four strains. The average nucleotide identify (ANI) values between any two strains ranged from 99.05% ~ 99.71%, and the proportion of the total base length of collinear blocks accounts for the total gene length of corresponding genome was all more than 93.82%. Then, we performed a search for genomic islands, prophage sequences, the gene clusters macromolecular secretion systems, type III secreted effectors and other virulence factors in these strains, which provided detailed comparison results of their presence and distinctive features compared to the reference strain GMI1000. Among them, the number and types of T2SS gene clusters were different in the four strains, among which RS650 included all five types. T4SS gene cluster of RS639 and RS647 were missed. In the T6SS gene cluster, several genes were inserted in the RS639, RS647, and RS650, and gene deletion was also detected in the RS642. A total of 78 kinds of type III secreted effectors were found, which included 52 core and 9 specific effectors in four strains.

**Conclusion:**

This study not only provided the complete genomes of multiple *R. pseudosolanacearum* strains isolated from a new host, but also revealed the differences in their genomic levels through comparative genomics. Furthermore, these findings expand human knowledge about the range of hosts that *Ralstonia* can infect, and potentially contribute to exploring rules and factors of the genetic evolution and analyzing its pathogenic mechanism.

**Supplementary Information:**

The online version contains supplementary material available at 10.1186/s12864-024-10087-7.

## Background

*Ralstonia solanacearum* species complex (RSSC) is a phytopathogenic bacterium belonging to β subdivision of the Proteobacteria [1] having a wide geographical distribution ranging from tropical, subtropical and warm temperate regions. RSSC is also known to infect more than 397 plant species distributing in 78 botanical families [2], including many solanaceous crops such as tomato, potato, and pepper resulting in significant losses during crop cultivation [3]. The plant resistance to bacterial wilt often breaks down due to the large genetic and phenotypic diversity within RSSC and the movement of exotic strains into new regions [4, 5]. At present, we still lack an efficient and environmentally friendly control measure for most of the crops bacterial wilt.

Historically, RSSC strains have been classified into biovar and race based on their carbon utilization patterns and host range [6, 7], but these classifications are neither predictive nor phylogenetically meaningful. Due to its extensive genetic diversity [8], *R. solanacearum* is often referred to as a species complex. Since 2014, RSSC was divided into three species, *R. solanacearum*, *R. pseudosolanacearum* and *R. syzygii*. *R. solanacearum* strains were mostly from the America, *R. pseudosolanacearum* strains were mostly from Asia and Africa, *R. syzygii* strains were from Indonesian archipelago [9–11].

Strains are further sub-classified into “sequevars”, based on sequence variation in the endoglucanase (*egl*) partial gene and other reference genes. Up to now, RSSC strains have been divided into 71 sequevars [12]. However, a genomic meta-analysis indicates that several of the *R. pseudosolanacearum* sequevars exhibit polyphyletic characteristics [13]. Moreover, until recently, there has been a lack of a standardized protocol for assigning sequevars, so the imprecision of *R. pseudosolanacearum* sequevars could be due to differences in sequence trimming, alignment, and thresholds between research groups. Consequently, it is advisable to exercise caution when interpreting sequevars in *R. pseudosolanacearum*. Nevertheless, the Chinese RSSC strains belong mainly to *R. pseudosolanacearum* strains, which possess high level of phylogenetic diversity, comprising a total of 16 sequevars (12~18, 34, 44, 45, 48, 54~57 and 14 M) from different host crops that are currently known [14–16]. Recently, new sequevars 70 and 71 were identified and isolated from *Bidens pilosa* and potato [12]. Interestingly, *R. pseudosolanacearum* isolated from the same host showed high genetic diversity. For example, tobacco can be infected by at least 10 sequevars (1, 13~18, 34, 44, 54, 55) [16, 17]. The diversity of *R. pseudosolanacearum* isolated from tobacco is negatively affected by altitude, which means that the differences are smaller in plateau areas [16] possible be related to adapting to low temperatures. In addition, the prevalence and virulence were different in the different sequevars of RSSC isolated from the same hosts. The sequevar 1 isolated from potato is the most prevalent and as a highly or medium virulence strain, because 91% of 123 potato RSSC isolates from 13 provinces belong to sequevar 1. Other sequevars 13~17 and 14 M belong to the low virulence strains in potato [14]. The generation of the new sequevars was often related to different geographical locations, ecological environments, cultivation measures, and irrigation water [16, 18, 19]. However, it seems that little attention has been paid to the genetic diversity of RSSC isolated from the same crop in the same field.

Our previous studies showed that *R. pseudosolanacearum* isolated from the same host plant could include several sequevars with different virulence [20–22]. In 2009, Ramesh et al. reported the pathogen (strain RS-09–190) of the sunflower bacterial wilt in India, which was identified as *Ralstonia pseudosolanacearum* and biovar 3 [23]. However, the genomes of any strain isolated from sunflower have not been reported so far. Cultivated sunflower (*Helianthus annuus*) is the fourth major oilseed crop in China. In May, 2020, the pathogens of sunflower bacterial wilt were identified as *R. pseudosolanacearum* including sequevars 13, 14, 17 and 54 [20]. In the current study, we performed a comprehensive comparative analysis of the genomes of four *R. pseudosolanacearum* strains belonging to four different sequevars. Four strains were isolated from sunflower in the same field but showed different pathogenicities to the same crop cultivar and different host range. In this study, we have analyzed genomic elements that may be related to the potential pathogenicity of populations.

## Results

### Four strains show different pathogenicity to the same cultivars

Inoculated plants began to wilt at 5 days post inoculation (dpi). The number of diseased plants was stable at 35 dpi. The results of  statistical analysis indicated that the pathogenicities of four strains RS639, RS642, RS647 and RS650 towards the same cultivars varied. Four strains were highly pathogenic to Dongqie, with disease incidence (DI) values ranging from 93.33% to 100%. RS639 and RS642 were highly pathogenic to Xinxing 101, with DI values of 80% and 91.11%, respectively, while RS647 and RS650 exhibited low pathogenic to Xinxing 101, with DI values of 14.33% and 26.67%, respectively. RS639, RS642 and RS647 were moderate pathogenic to three eggplant cultivars, with DI values from 20% to 62.22%, RS650 only could infect the Bailong cultivar, with DI value of 15.56%. RS639 and RS642 were highly pathogenic to three pepper cultivars, with DI values from 86.67% to 100%, RS647 was highly pathogenic to two pepper cultivars Yueshu No.2 and Yuehong No.3, with DI values of 66.67% and 80%, respectively, but low pathogenic to Huifeng No.2 cultivar with DI value of 6.67%. In addition, RS639 was highly pathogenic to tobacco, RS650 was low pathogenic to tobacco, and RS642 and RS647 were not pathogenic to tobacco. RS639, RS642 and RS650 were low pathogenic to *R. kaempferiae* with the DI value from 3.33% to 33.33%, while RS647 was not pathogenic to *R. kaempferiae* (Table 1)*.* Those results indicated that the four strains were different in pathogenicities and host ranges.

**Table 1:** Pathogenicity of four strains isolated from sunflower

Isolates	Average disease incidence (%)									
	Tomato (Dongqie)	Tomato (Xinxing 101)	Eggplant (Qingqie)	Eggplant (Nongfu No.2)	Eggplant (Bailong)	Pepper (Huifeng No.2)	Pepper (Yuehong No.3)	Pepper (Yueshu No.2)	*Nicotiana tabacum*	*Rhizoma kaempferiae*
RS639	100.00	80.00	20.00	20.00	62.22	86.67	100.00	100.00	86.67	3.33
RS642	100.00	91.11	53.33	20.00	62.22	100.00	100.00	100.00	0.00	33.33
RS647	93.33	14.33	20.00	20.00	40.00	6.67	80.00	66.67	0.00	0.00
RS650	93.33	26.67	0.00	0.00	15.56	26.67	73.33	46.67	6.67	13.33

### Sequencing, assembly and general features of four strains

**Fig. 1:**
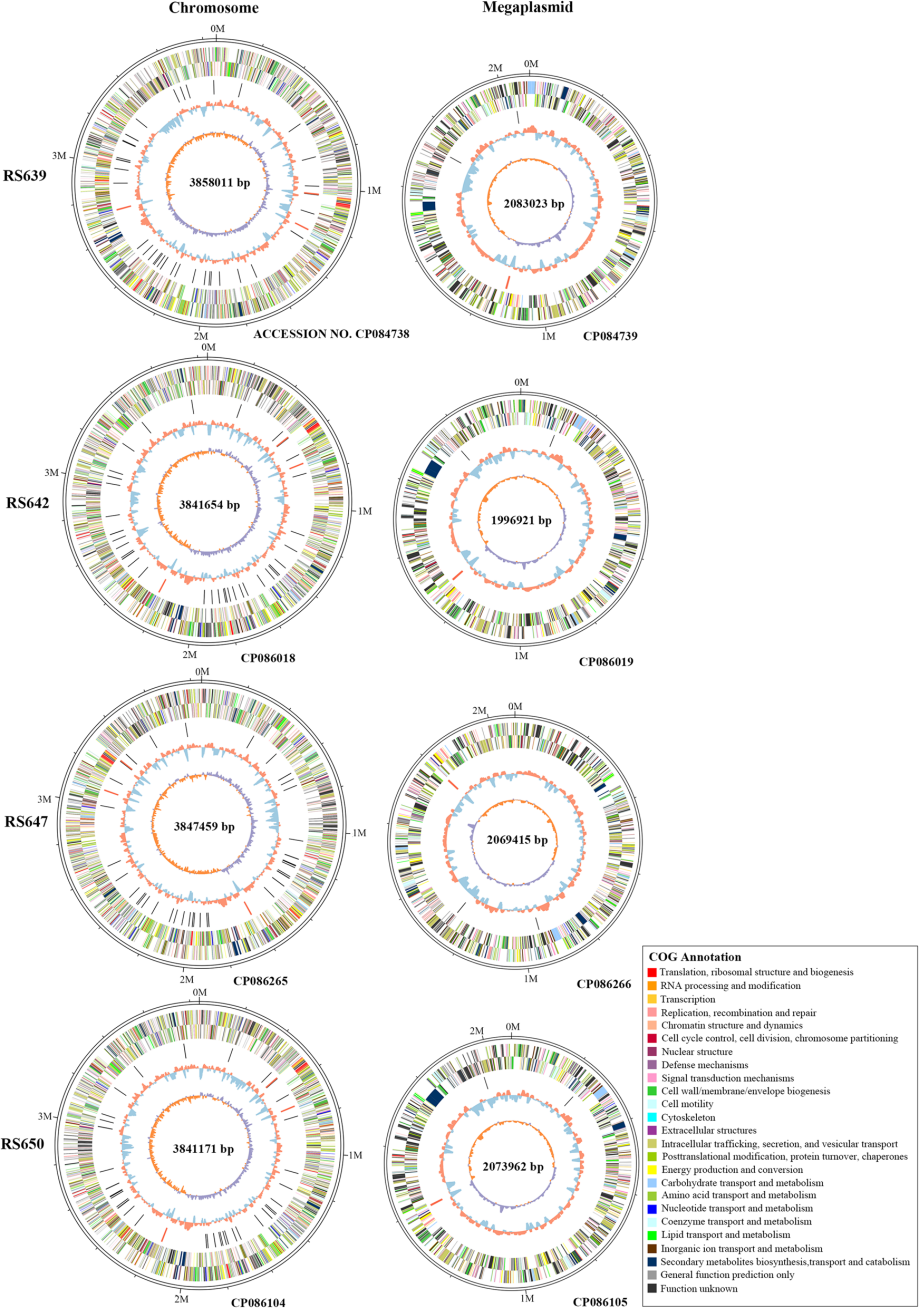
The circular maps of the four genomes chromosome (top), megaplasmid (bottom). The circles from outer to inner represent the genome size, CDS (different colors represent different functional classifications), ncRNA, GC ratio (green, outward means GC ratio of the region is higher than average GC ratio; red, inward means GC ratio of the region is lower than average GC ratio), GC skew (light green represents a region with G content greater than C, pink represents a region with C content greater than G)

Four high-quality genome assemblies were generated for *R. pesudosolanacearum* strains RS639, RS942, RS647, and RS650 using a combination of PacBio long read data and Illumina short read data. As a result, a total of 1.3 Gb ~ 1.4 Gb polymerase reads from a 20 kb library were generated by Single Molecule Real-Time (SMRT) sequencing. After removing adapters and low-quality or ambiguous reads, we obtained 1.3 Gb ~ 1.4 Gb (~ 222 × to 251 ×) subreads for four complete genome assemblies. The genomes of four strains all consisted of a circular chromosome and a circular megaplasmid; no small plasmid was found in any four strains (Fig. 1). The sequences of four genomes were assembled into different sizes, of which the largest genome was RS639 (5,941,034 bp with 66.85% GC content), and the smallest genome was RS642 (5,838,575 bp with 67% GC content) (Table 2). A total of 5304, 5259, 5306 and 5300 genes were annotated by GeneMarkS-2 + software, among which 5056, 5002, 5057 and 5079 genes encoded complete coding sequence (CDS) in the genome of RS639, RS642, RS647 and RS650, respectively. Other than the protein coding genes, the four genomes also encodes 12 rRNAs and 59, 58, 57 and 58 tRNAs. In addition, 173, 183, 176 and 147 pseudogenes were identified in the whole genome of RS639, RS642, RS647 and RS650, respectively.

**Table 2:** Genomic features of four strains isolated from sunflower

Strains	RS639	RS642	RS647	RS650
Coverage	222	230	234	251
Genome size (bp)	5941034	5838575	5916874	5915133
Chromosome (bp)	3858011	3841654	3847459	3841171
Megaplasmid (bp)	2083023	1996921	2069415	2073962
GC content (%)	66.85	67	66.86	66.91
Predicted CDSs	5056	5002	5057	5079
rRNAs	12	12	12	12
ncRNAs	4	4	4	4
tRNAs	59	58	57	58
Genomic islands	24	17	22	21
Prophage regions	3	4	3	6
CRISPRs	1 (questional)	3(1 questional, 2 credible)	2(questional)	1(credible)
Transposon	11	14	19	9

**Fig. 2:**
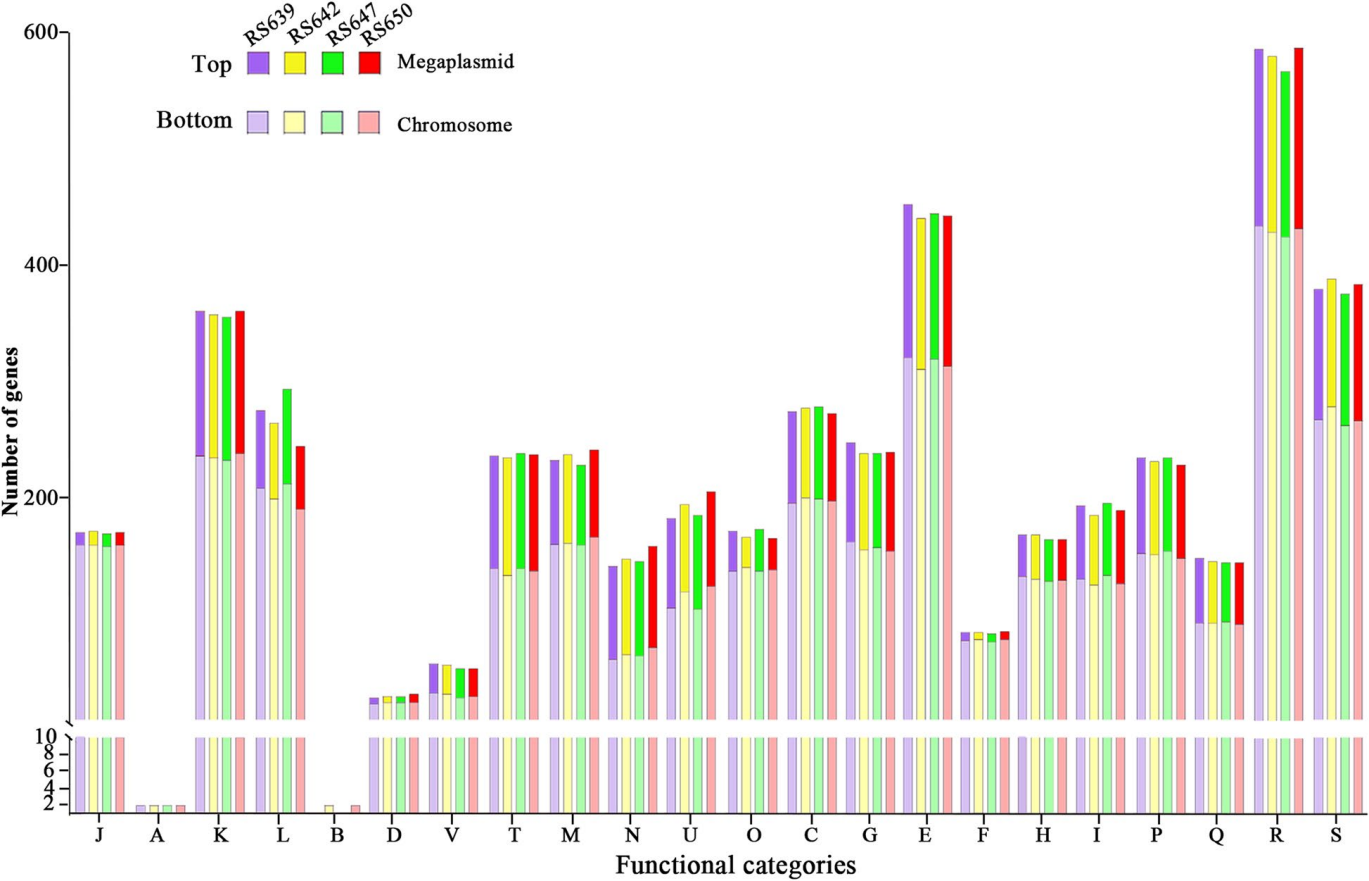
The distribution of genes with COG functional categories in the chromosomes and megaplasmids of four strains isolated from sunflower. The upper part of the bar represents the number of genes in the megaplasmids, while the lower part represents the number of genes in the chromosomes. J: Translation, ribosomal structure and biogenesis; A: RNA processing and modification; K: Transcription; L: Replication, recombination and repair; B: Chromatin structure and dynamics; D: Cell cycle control, cell division, chromosome partitioning; Y: Nuclear structure; V: Defense mechanisms; T: Signal transduction mechanisms; M: Cell wall/membrane/envelope biogenesis; N: Cell motility; Z: Cytoskeleton; W: Extracellular structures; U: Intracellular trafficking, secretion, and vesicular transport; O: Posttranslational modification, protein turnover, chaperones; C: Energy production and conversion; G: Carbohydrate transport and metabolism; E: Amino acid transport and metabolism; F: Nucleotide transport and metabolism; H: Coenzyme transport and metabolism; I: Lipid transport and metabolism; P: Inorganic ion transport and metabolism; Q: Secondary metabolites biosynthesis,transport and catabolism; R: General function prediction only; S: Function unknown

Functional annotation successfully classified 2652 ~ 2682 genes in the chromosome into 22 Clusters of Orthologous Groups (COG) categories, and 1057 ~ 1088 genes in the megaplasmid into 20 COG categories in the four genomes (Fig. 2, Table S1). Comparison of the genome distribution of genes in different COG function categories showed the number of gene contents on chromosome were more than that of megaplasmid, even two types of functional categories, RNA processing and modification, Chromatin structure and dynamics, were missed from the megaplasmid. But the megaplasmid had more genes encoding cell motility than the chromosome. In the four genomes, except for the genes predicted to have general or unknown functions (944 ~ 972 genes), the largest group of genes were all in Amino acid transport and metabolism roles (441 ~ 453 genes) and followed by 356 ~ 361 genes responsible for transcription, 273 ~ 279 genes involved in replication, recombination and repair, and 245 ~ 294 genes involved in energy production and conversion. Besides, 355, 361, 358 and 360 genes in RS639, RS642, RS647 and RS650 respectively were homologous with those genes in the Virulence Factors of Pathogenic Bacteria (VFDB)database (Table S2). There were 1078, 1084, 1058 and 1082 genes in RS639, RS642, RS647 and RS650 respectively, which were homologous with those genes in the Pathogen Host Interactions (PHI) database (Table S3). However, the classification results of these functional genes showed no obvious differences among the four strains.

### Comparative genome analyses of strains to other *R. solanacearum* species complex strains

**Fig. 3:**
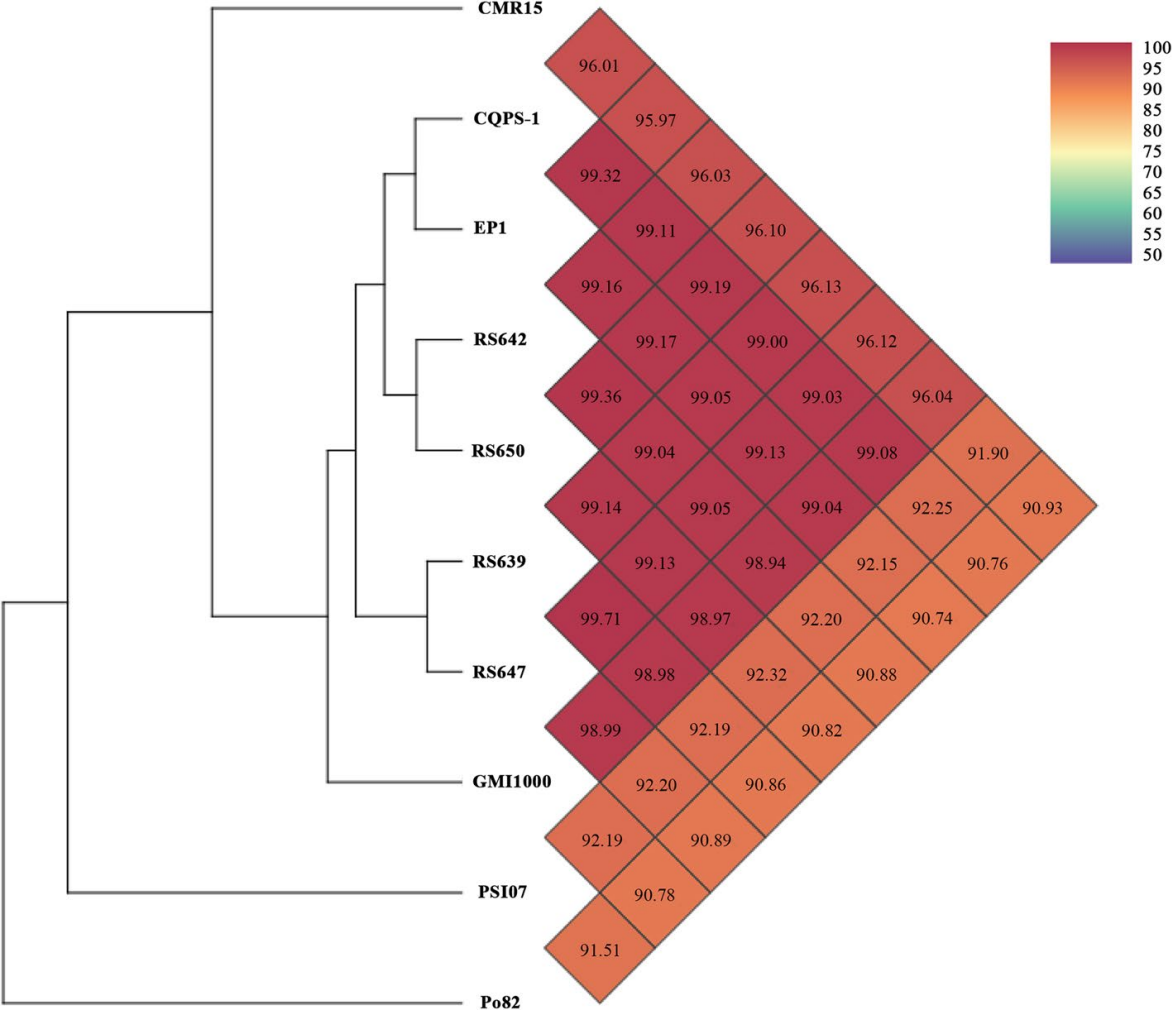
Pairwise average nucleotide identity (ANI) comparisons of whole genomes in four strains isolated from sunflower and other 6 strains. ANI values are also coded in color as explained in the legends. ANI is calculated based on fragment size of 500 bp

**Fig. 4:**
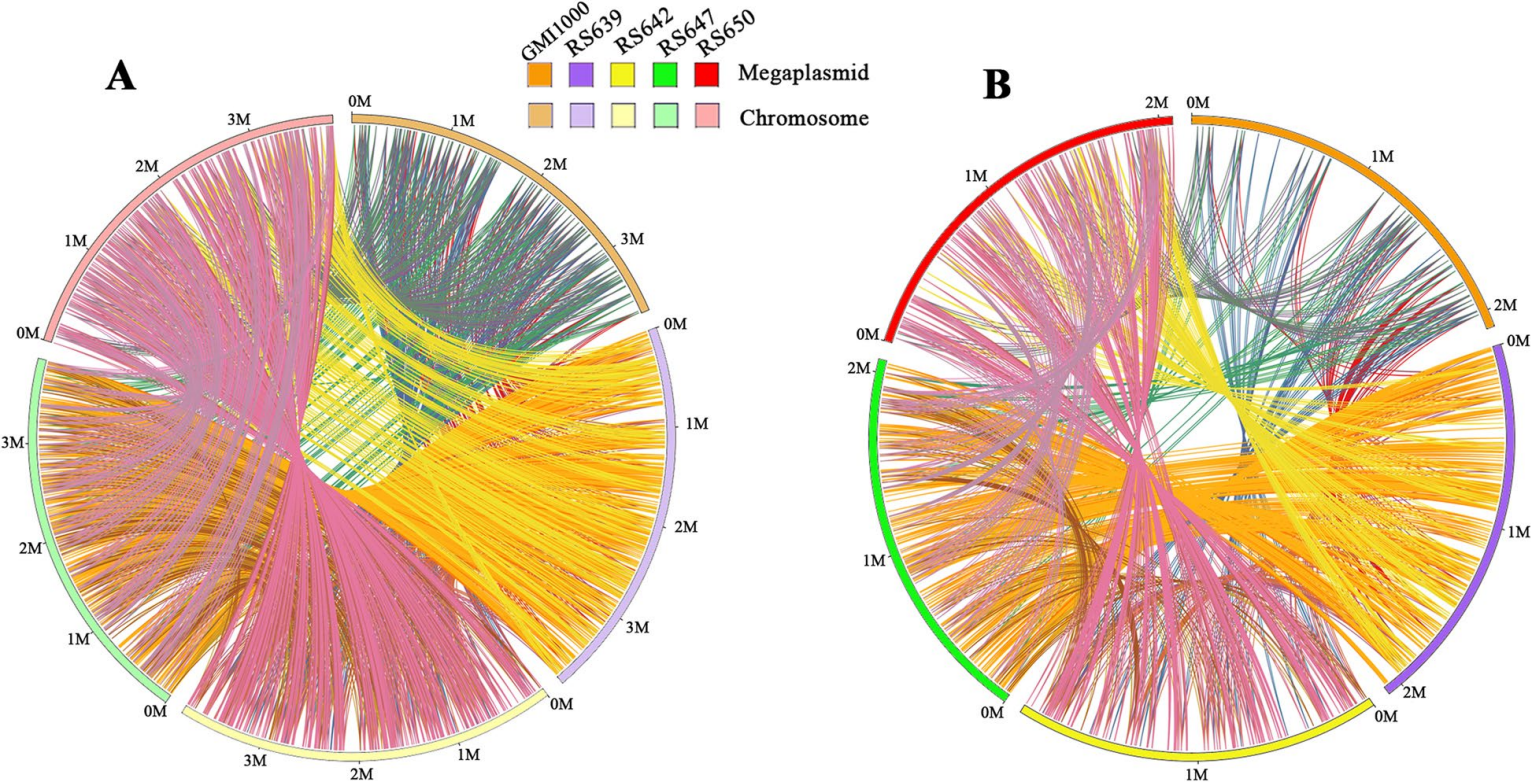
Nucleic acid co-linearity of the chromosomes (**A**) and megaplasmids (**B**) in the four strains isolated from sunflower. These sequences of RS639, RS642, RS647 and RS650 are ordered according to that of the reference strain GMI1000 based on MUMmer 3.1. The pairwise nucleic acid sequence of two alignments is marked in the coordinate diagram according to its position information

The similarity of genome sequences between four strains and six *R. solanacearum* species complex strains was measured by Orthologous Average Nucleotide Identity Tool (OAT). The results showed that RS639, RS642, RS647, and RS650 were closely related with each other with the pairwise average nucleotide identify (ANI) values ranging from 99.05% ~ 99.71%, grouping together with other three *R. pseudosolanacearum* strains (Fig. 3). Within the clade, strain RS639 was almost equally similar to RS647, with ANI value of 99.97%, and RS642 was almost equally similar to RS650, with ANI value of 99.36%. To further evaluate the genome evolution of these four strains, the genome sequences of four strains and the reference strain GMI1000 were aligned using the MUMmer program (Fig. 4). The results showed that the proportion of the length of collinear blocks between any two of four genomes accounts for the length of corresponding genome was all more than 93.82%. RS642 was the most co-linear with RS650, and the full length of collinear blocks accounts for 97.44% and 96.97% in the full genome of RS642 and RS650, respectively. Moreover, further analysis showed that these values of the proportion were 91.32% ~ 95.91% in the chromosomes and 89.64% ~ 98.79% in the megaplasmids. RS650 (91.32%) was the least matched with strain RS647 (93.3%) in chromosomes, and RS647 was the least matched with strain RS642 (89.95%). In brief, there were some inversion fragments and dissimilarities among these strains, both in the chromosomes and megaplasmids.

**Fig. 5:**
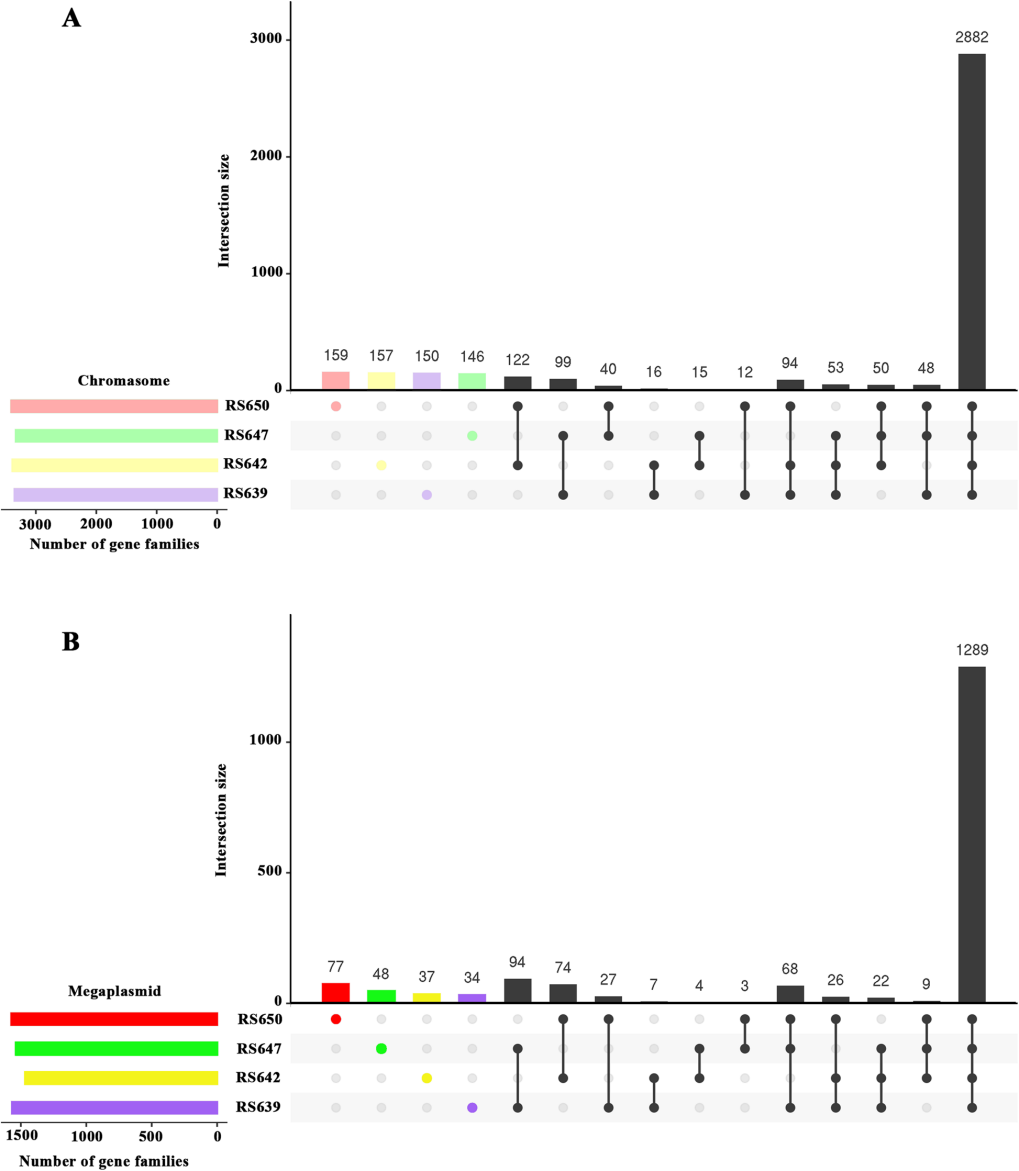
UpSet diagram showing deduced gene families of four strains isolated from sunflower by pan-genome. Values are calculated by OrthoMCL analyses with the parameters: *P*-value Cut-off = 1 × 10^−5^, Identity Cut-off = 90%, Percent Match Cut-off = 80. The combination matrix identifies the intersections, while the bars above to it encode the numbers of gene families of each intersection

Then, we performed pan-genome analyses on chromosomes and megaplasmids from four strains (Fig. 5, Table S4). A total of 4404 and 1819 gene homolog families were identified across chromosomes and megaplasmids, respectively. Interestingly, 146 ~ 159 specific gene families (contained 150 ~ 160 genes) were found in the chromosomes and 34 ~ 77 specific gene families (contained 34 ~ 78 genes) in the megaplasmids from four strains. Among them, RS650 had the most strain-specific gene families, which was true in both chromosome and megaplasmid. In the results of correlation analysis, RS650 and RS642 shared the most gene families in the chromosomes, and RS647 and RS639 shared the most gene families in the megaplasmids. The final core genome comprised 2882 gene families in the chromosomes and 1289 gene families in the megaplasmids, which were shared by all compared strains. In addition, we also provided the results of gene family correlation among all strains.

### Genomic islands, CRISPR and prophage sequences prediction

One of the evidence for horizontal origins in bacteria is the existence of genomic island (GI) [24]. A total of 84 GIs were predicted in the four strains. In strain RS639, the total length of GIs was 458,852 bp, distributed in 24 GIs. Three type III effectors were located in the GIs, which were RipS4, RipM and RipP1. In strain RS642, the total length of GIs was 272,511 bp, distributed in 17 GIs. Type III effector RipAX2 located in the GIs. In strain RS647, the total length of GIs was 395,031 bp distributed in 22 GIs. Type III effector RipP1 located in this GIs. In strain RS650, the total length of GIs was 392,884 bp, distributed in 21 GIs. The same as RS647, there was only type III effector RipP1 located in the GIs (Table S5).

Prophages in bacterial genome also suggest the occurrence of horizontal gene transfer events. They frequently encoded virulence genes and were major contributors to the genetic individuality of the strains [25]. In total, 15 prophage-like elements were identified in the four strains using PHASTER software (Table S6). 14 were found to be integrated into chromosomes, and only 1 into megaplasmid of RS650. The sizes of these prophage-like elements ranged from 1.5691 to 4.0999 kb, and contained 21 to 51 genes. The GC contents of these prophage-like elements were 62.61% to 66.89%, which were all lower than that of their corresponding genomes. The blast results by NCBI database showed that 15 prophage-like elements were compassed in five types. Among them, the largest number (9) of prophage sequences containing in all four strains were matched to the partial sequence of Vibrio phage VHML (NC_004456), and the coverages were 37%~89%, identities were 97.3% ~ 96.8% between them and RS639-Prophage_1-chr. In addition, we found that the partial sequences from *Burkholderia* Phage PHIE 125 (NC_003309), Entero Bacteria Phage SF6 (NC_005344) and Entero Bacteria Phage SFV (NC_003444), were existed in the strains RS642, RS647 and RS639, respectively. One partial sequences of *Ralstonia* phage phiRSA1 (NC_009382) were all contained in the strains RS639, RS642 and RS650, which shared the coverages 52% ~ 79% and identities 93.6 ~ 95.3% between them.

### Comparison analyses of secretion systems

**Fig. 6:**
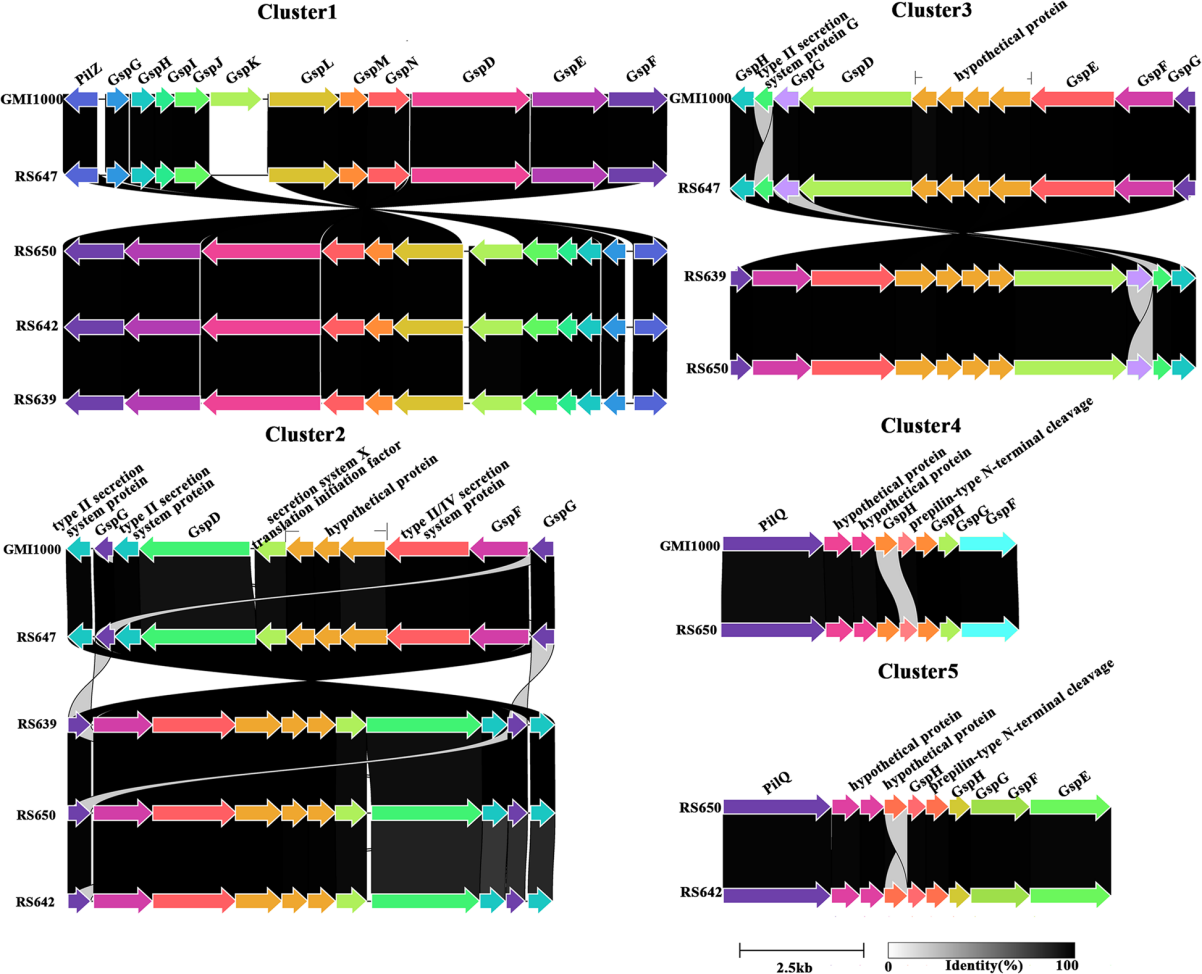
Genetic organization of T2SS gene clusters in four strains isolated from sunflower. Linkages are drawn between homologous genes. The synteny and global amino acid identity are visualized with Clinker, and aesthetics are adjusted in Adobe Illustrator

Secretion systems are essential for bacteria to adapt to the wide range of environmental conditions [26]. In this study, four important secretion systems (type II, III, IV and VI) were compared between four strains and GMI1000. GMI1000 possessed four gene clusters of type II secretion systems (T2SS). Cluster-1 was the orthodox system encoded by 12 genes in the chromosome (RS_RS15650 ~ RS_RS15595). The other three T2SS were unorthodox systems. Eight core genes and three hypothetical genes, seven core genes and four hypothetical genes, and six core genes and two hypothetical genes were possessed in Cluster-2 (RS_RS11590 ~ RS_RS11540), Cluster-3 (RS_RS17870 ~ RS_RS17820) and Cluster-4 (RS_RS19435 ~ RS_RS19400), respectively. A total of five T2SS gene clusters were found in four strains (Fig. 6, Table S7). Strains RS639 and RS647 possessed three T2SS gene clusters, which were similar to Cluster-1, Cluster-2 and Cluster-3 in GMI1000. Except that GspK encoded by LGV81_15980 gene was a pseudogene, the genes of the three clusters were highly conserved and shared a high similarity with strain GMI1000 (coverages 97.2% ~ 100%, identities 98.4% ~ 100%). Strain RS647 possessed three T2SS gene clusters, two T2SS gene clusters were similar to Cluster-1, Cluster-2 in GMI1000, with coverages range from 93.2% to 100%, and identities range from 84.4% to 99.9%. The third gene cluster, named cluster-5, was lacking in GMI1000, and it possessed seven core genes and two hypothetical genes. Strain RS650 possessed five T2SS gene clusters, among which four T2SS gene clusters were similar to that in GMI1000, with coverages range from 95.4% to 100%, and identities range from 96.7% to 99.9%. Cluster-5 was similar to that in RS642.

Type III secretion system (T3SS) injecting effectors into plant cell, is important for the pathogenicity of *R. solancearum* complex species [27]. T3SS is composed of hypersensitive response and pathogenicity (*hrp*) gene clusters present in the megaplasmid. So, we further analyzed the *hrp* gene cluster in the four strains and GMI1000 (Table S7). These *hrp* gene clusters spanned 2.8603 kb ~ 2.9626 kb, and all were located in the megaplasmid and composed of 30 *hrp* genes in the four strains, which were consistent with these characteristics of GMI000 strain. Except the gene *SctC* in strain RS650 was a pseudogene, other genes were very conservative (coverages 95.9% ~ 100%, identities 98.9% ~ 100%) in the four strains. These results indicated that the discrepancy in virulence between the four strains was not likely due to T3SS structural genes. This showed that the T3SS structural differences of the four strains were not particularly obvious, which might not be the cause of phenotypic differences.

**Fig. 7:**
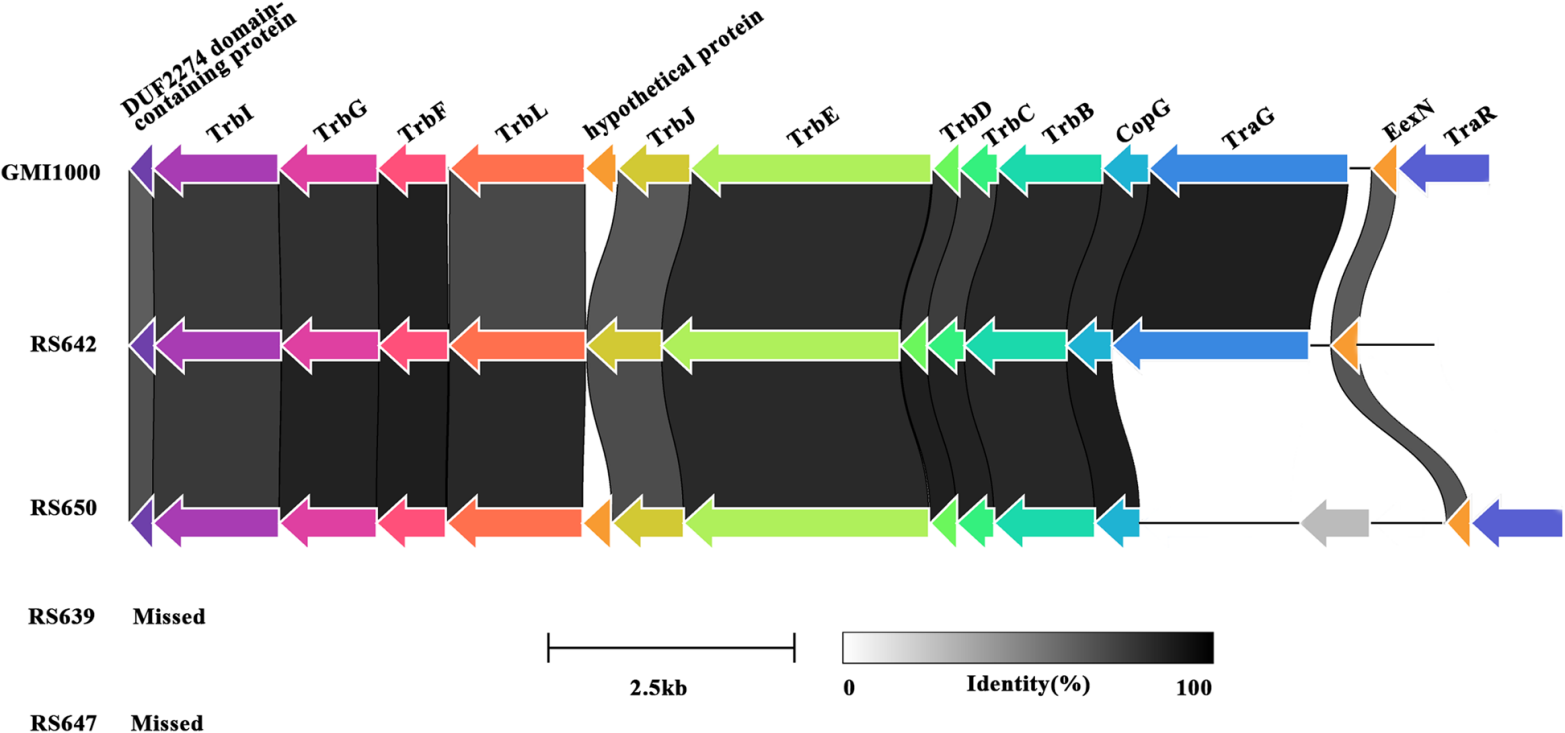
Genetic organization of T4SS gene clusters in four strains isolated from sunflower. Linkages are drawn between homologous genes. The synteny and global amino acid identity are visualized with Clinker, and aesthetics are adjusted in Adobe Illustrator

Similarly, we analyzed type IV secretion system (T4SS) gene cluster between the four strains and GMI1000 (Fig. 7, Table S7). No T4SS gene cluster was found in the strains RS639 and RS647. There were 13 homologs between RS642 and GMI1000, while RS642 lacked the homologous genes trbL and trbJ, and owns a pseudogene of traR. There were 14 homologs between RS650 and GMI1000, while conjugal transfer protein TraG in RS642 was split into two pseudogenes (LGV82_09965 and LGV82_09975) by inserting a gene coding for IS5 family transposase (LGV82_09970). The T4SS genes among RS642, RS650 and GMI1000 showed similar gene rearrangements, and the similarities between these genes ranged from 65.1% to 88.2%.

**Fig. 8:**
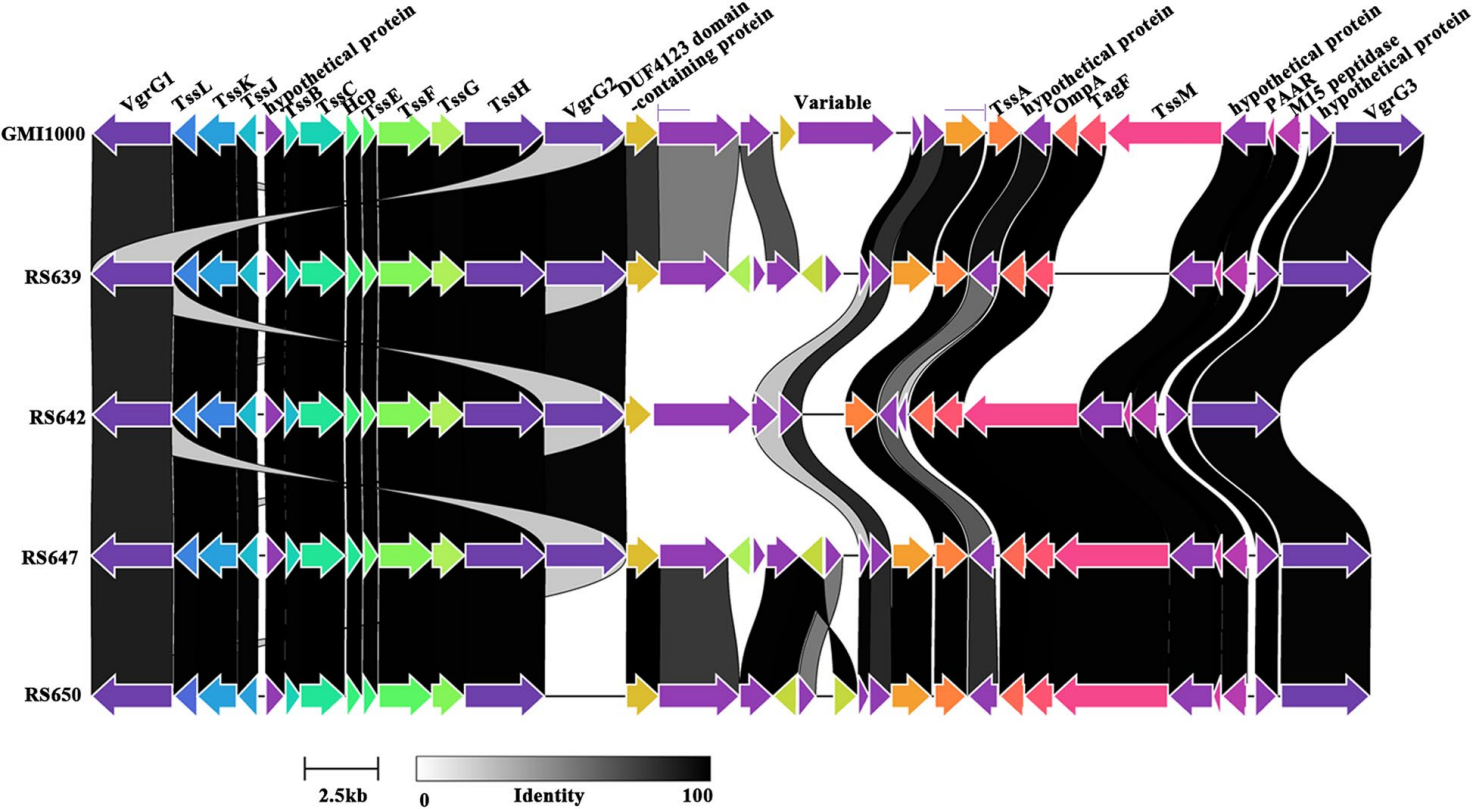
Genetic organization of T6SS gene clusters of four strains isolated from sunflower. Linkages are drawn between homologous genes. The synteny and global amino acid identity are visualized with Clinker, and the aesthetics are adjusted in Adobe Illustrator

Type VI secretion system (T6SS) widely exists in Gram-negative bacteria, and plays an important role in its inter-microbial competitiveness [28]. T6SS genes showed similar gene rearrangements. In strain GMI1000, the T6SS locus was located in the megaplasmid, which spanned a 45.457 kb region (RS_RS20795 ~ RS_RS20645) and included 31 genes (Fig. 8, Table S7). Among the four strains isolated from sunflower, the gene clusters of T6SS were all located in their megaplasmid, with the sizes of 40.542 ~ 43.695 kb, encoding 28 ~ 33 genes. The results of clinker comparison showed that the T6SS genes located between vgrG1 and vgrG2, and between vgrG3 and GH10 (glycoside hydrolase family 10 protein) were relatively conservative, and while the genes between DUF4123 domain-containing protein and GH10 were much variable, which was consistent with the previously reported T6SS clusters in the other RSSC. It was worth noting that the core genes tssM and GH10 of T6SS were pseudogenes in the strains RS639 and RS642, respectively. In addition, there were different numbers (3 ~ 8 genes) of genes in the variable regions of the four strains, which were mainly nonfunctional proteins or genes encoding the transposase.

To sum up, except for the relatively conservative T3SS gene cluster, there were certain differences among type II, IV and VI gene clusters in the four strains isolated from sunflower.

### Repertoire and comparative analysis of T3Es of four strains

**Fig. 9:**
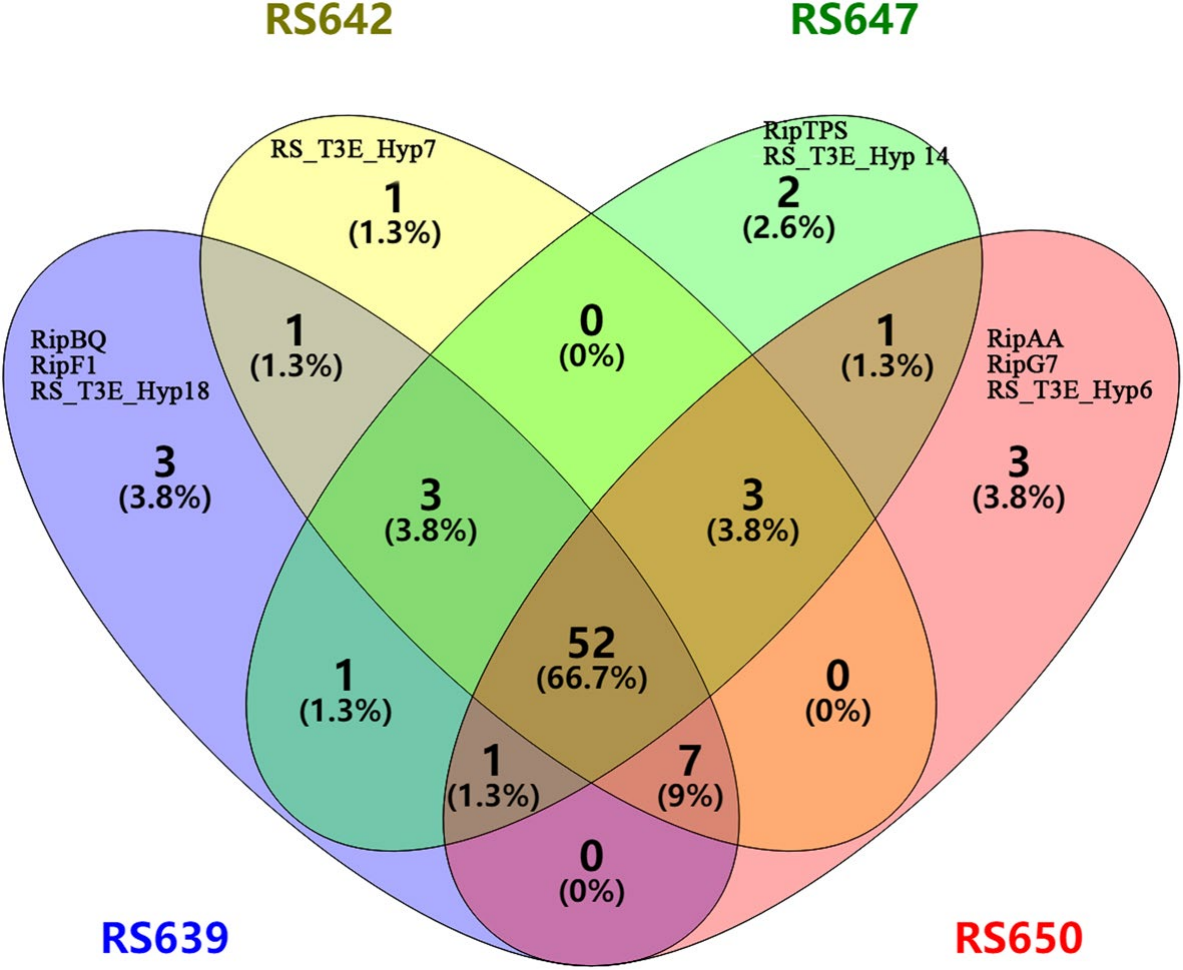
T3Es distribution of four strains isolated from sunflower. The overlapping sections indicate shares numbers of T3Es, and the numbers indicate the number of T3Es in this area

We identified the type III secreted effectors (T3Es) according to the RalstoT3E database [29]. The number of T3Es in the genome of strains RS639, RS642, RS647 and RS650 were 65, 65, 62 and 66, respectively. A total of 78 kinds of T3Es and 52 (66.7%) were conserved and belonged to the core T3Es among the four strains whereas 9 T3Es (11.5%) appeared to be strain-specific (Fig. 9, Table 3).There were 3 (RipBQ, RipF1 and RS_T3E_Hyp18), 1(RS_T3E_Hyp7), 2 (RipTPS, RS_T3E_Hyp 14) and 3 (RipAA, RipG7 and RS_T3E_Hyp6) strain-specific genes located in strains RS639, RS642, RS647 and RS650, respectively. In addition, RipD was only absent in strain RS639, RipC2 was only absent in strain RS642, and five effectors, RipA2, RipA3, RipAX2, RipG6 and RipT, were only absent in strain RS647, RipAW, RipE1 and RipV1 were only absent in strain RS650. Besides, 2 (RipS4 and RipU), 2 (RipAV and RipJ), 1 (RipW) pseudogenes were existed in strains RS639, RS642, RS647, respectively, but none in strain RS650. This confirms that a majority of T3Es are widely conserved in the four strains but also shows that the strain repertoires are also diversified. Then, we compared the genes of T3Es of four strains to GMI1000. Compared with GMI1000, RipG4, RipQ, RipS1, RipAG, RipAH, RipAX1, RipBJ and RipBO were absent, but RipAL, RipBA, RipBK, RipBQ, RipBP, RS_T3E_Hyp6, RS_T3E_Hyp7, RS_T3E_Hyp14 and RS_T3E_Hyp18 were added in the four strains. The sequence similarities of RipG6, RipG7, RipU, and RipY in four strains were all below 90%, ranging from 21.8% to 81.4%. It's noteworthy that RipAD in strains RS639, RS642 and RS650 shared more than 98% similarities to the counterparts of the strain GMI1000, but in strain RS647 only shared 82.8% similarity because of 63 base pair random insertion. Importantly, RipAA, RipAF1, RipAI, RipAW, RipB, RipC1, RipG3 and RipT had highly similarities but lowly coverages to the counterparts of the strain GMI1000. These results showed that there were differences in the types and similarities of T3Es between four sunflower strains and strain GMI1000, which might provide data and reference for the study of specific effectors related to host range.

**Table 3:**
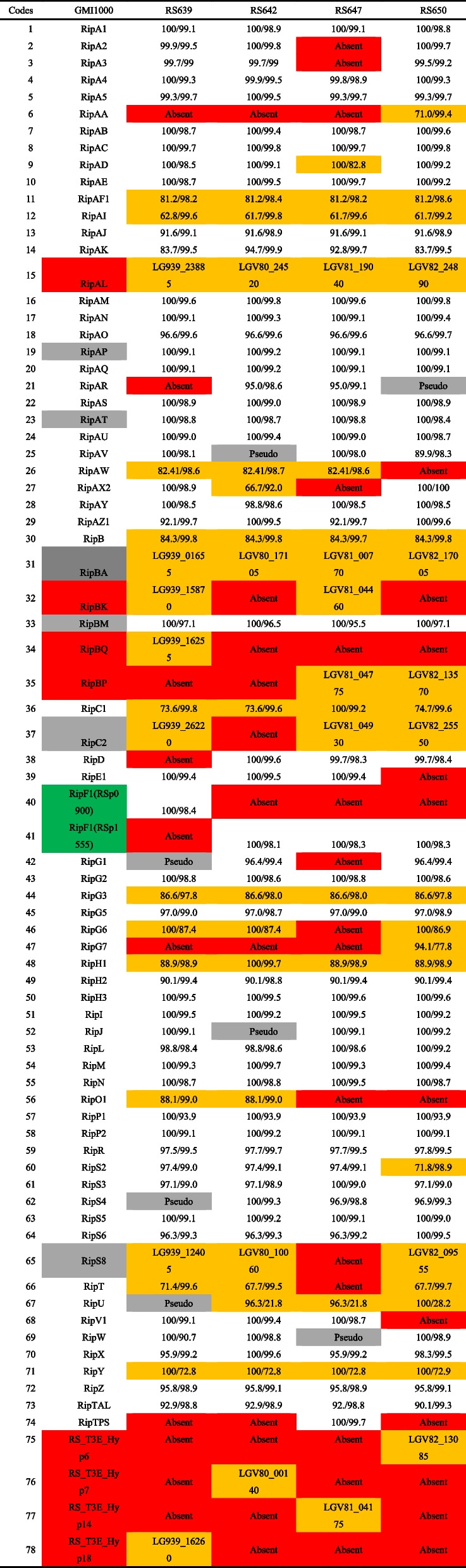
Comparative analysis of T3Es genes between four strains isolated sunflower and strain GMI1000

Gray mark indicated that the gene was a pseudogene; Red gene was missing; Green gene was a multi-copy gene; White gene was a single copy gene; Yellow gene was specific in part strain, or the coverage/ identity less than 90%. The number in the table was coverage/identity (%)

### Other virulence factors

In addition to the above genes, there are many other genes involved in the pathogenicity of RSSC. We also compared 42 virulence genes associated key regulatory factors, Exopolysaccharide (EPS) biosynthesis genes, elicitins, biofilm and other genes required for infecting host or surviving stressful environments and compounds between these four strains and GMI1000. The results showed that these genes were highly conserved, and the similarity with GMI1000 was 99% ~ 100% (Table S8).

## Discussion

In 2020, we first reported sunflower bacterial wilt in China, and identified the pathogen as *R. pseudosolanacearum* race 1, biovar 3, and including four sequevars (13, 14, 17 and 54) [20]. In this study, the pathogenicities of four sequevars *R. pesudosolanacearum* strains of sunflower bacterial wilt were carried out on five hosts including eight cultivars, and the novel complete genome of four strains were firstly sequenced using a combination of PacBio and Illumina HiSeq2000 sequencing technologies, and performed genome-wide comparisons between these strains and the reference strain GMI1000. Our results further demonstrated that *R. solanacearum* species complex can continuously produce new bacterial strains through genomic mutation, gene deletion/insertion, and genomic rearrangements. In short, the results provide an important basis for gaining insight into the variance of virulence factors and genome diversity of RSSC.

Over 5000 strains of RSSC from more than 88 regions were assigned to over 70 sequevars, and 36 sequevars were reported in *R. pesudosolanacearum* [2]. The pathogenicities of strains with the different sequevars isolated from the same host were different [12, 30], but the specific reasons have not been systematically explored. In addition, most studies on the pathogenicity and host range of RSSC focused on isolating strains from different crops and different geographical locations, while few studies paid attention to strains isolated from the same crop and the same geographical location[31, 32]. We also found that four strains RS639, RS642, RS647 and RS650 isolated from sunflowers in the same area at the same time also had different pathogenicity to the same or different cultivars and different host ranges. Importantly, RS650 could not infect two eggplant varieties, Qingqie and Nongfu No.2, but the other three strains could infect them; RS642 and RS647 could not infect *N. tabacum*, but RS639 and RS650 could. In addition, the pathogenicity of the four strains to tomato (Xinxing 101) and pepper (Huifeng No.2) was significantly different. These findings may be of reference value for us to study how strains change hosts or regulate pathogenic processes and related driving factors.

Genomes are a very useful resource to understand the mechanism of plant-pathogen interaction and explain for host range adaptation and pathogenicity of species [31]. The large genetic and phenotypic diversity within RSSC strains and the movement of exotic strains may be the main reason for the wide host range and difficulty in control of bacterial wilt at present [32]. Results of OAT analyses showed that four strains were more similar with two phylotype I strains (EP1, GMI1000, CQPS-1) (ANI > 98.94%) than strains from other phylotypes. According to the taxonomic standard that strains with ANI > 95% are considered as belonging to the same species[33], so the four strains from sunflower shall belong to the same species, *R. pesudosolanacearum*, consistent with our previous results[20]. The evolutionary changes such as chromosomal rearrangements always accompanied by host restriction in bacteria [34]. Though the highest genome similarity exists between *R. pesudosolanacearum* strains, but large amount of gene rearrangement events were unveiled[35]. MUMmer analyses showed inversions and gene deletion/insertion events were also found when comparing four genomes, and the chromosomes and plasmids of all four strains contained a number of strain-specific genes, which indicated the four strains were experiencing highly dynamic genome evolution even though they were collected in the same field and host. We also found that these genomes exhibited differentiation of genetic plasticity characterized by a large number of GIs and the various prophage sequences were helpful to expand the gene pools, especially the genes associated with virulence and resistance.

Macromolecular secretion systems could secrete proteins, DNA, or DNA–protein complex and be participated in key aspects of cell biology, such as nutrient acquisition, host-microbe or microbe-microbe interactions, motility, environmental adaptation, antibiotic resistance, and pathogenicity [26]. In this work, we explored the potential gene clusters of four secretion systems (II, III, IV and VI) that had differences in the genomes of four strains. T2SS is implicated in virulence factor secretions and colonization [36]. The number and types of T2SS gene clusters included in RS639, RS642, RS647, and RS650 were different, among which RS650 included all five types. Similarly, we have analyzed the T4SS that is known for translocation of genetic materials and effector proteins into host cytosol or other bacterial cells [37, 38], and the results showed that the T4SS gene cluster of RS639 and RS647 were deleted, and one gene was missed and one pseudogene was generated in RS642, two pseudogenes were generated due to the insertion of IS5 family transposase in RS650. Extensive studies have showed that T6SS could contribute to pathogenicity, host colonization, and mediate biofilm formation [39–41]. The main difference in the T6SS gene cluster of the four strains was the number of genes between the DUF4123 domain-containing protein and the glycoside hydrase family 10 protein. In this region, several genes (MFS transporter, IS5 family transporter, etc.) were inserted in the RS639, RS647, and RS650. Additionally, gene deletion was also detected in the RS642. Given the general important roles of these secretion systems in pathogens, these variations in the secretion systems gene cluster may likely cause changes in bacterial pathogenicity and the capability of host colonization.

According to our results, the *hrp* gene clusters of four strains were conserved compared with GMI1000, which is consistent with the previous report that *hrp* cluster was highly conserved among *R. pesudosolanacearum* [17]. The effectors (T3Es) secreted by T3SS play a critical role in bacterial virulence and determination of host range specificity [42]. RipP1, RipAA (formerly AvrA) and RipTPS triggered HR cell death and induced resistance to bacterial wilt in *Nicotiana tabacum* [43–45]. RipG7, the essential determinant of *R. solanacearum* strains for virulence on the legume plant *Medicago truncatula* [46]. RipAA and RipG7 only existed in strain RS650, RipTPS only existed in strain RS647. In addition, RipP1 existed in the genomic islands from four strains, indicating that it might have the potential to spread among different strains and even different species through horizontal transfer. In addition, several strain-specific effector proteins with no detailed functions were also found in this study, such as RipBQ, RS_T3E_Hyp18, RS_T3E_Hyp 14 and RS_T3E_Hyp6, so they might mediate some novel functional mechanisms, which also provided a good idea for our future research.

Bacterial genome variation refers to the phenomenon that occurs when bacteria reproduce and transfer genes, which is of great significance to the evolution and adaptation of bacterial population [47]. Bacterial genome variation mainly includes mutation, gene rearrangement and horizontal gene transfer. How can we explain such a great variety of genomic components in *R. pseudosolanacearum* strains isolated from the same plant species and grown on the same land? It is generally expected that these four isolated strains to be clonal or close to clonal, but in fact, a large number of strain-specific genes, low similarity genes and inversions or deletion/insertion events have been found in the four strains, and some core genes of the secretion system were mutated into pseudogenes in some strains; the potential foreign insertion genes have been found on different numbers and types of genomic islands and prophages in the four strains. Actually, it's not difficult to explain this phenomenon. First of all, bacteria have strong replication ability, which is easy to lead to base mutation of the genome. Secondly, it has been reported that the genome of RSSC has a mosaic structure, which makes it easy for RSSC to acquire exogenous DNA and lose preexisting genes [48, 49]. Finally, even in the same host and environment, each strain may face different providers of foreign genes in the different ecological niche, and there are certain factors and selection probability in the insertion of foreign genes.

In conclusion, these results revealed that in the process of interaction between pathogen and host plant, the genetic material of pathogen could be constantly changed to produce new strains, which may be an important way to maintain its pathogenicity and host range. The study on the differences in genomic components, genomic transfer, virulence genes and secretion system at the genomic level laid a foundation for studying the evolutionary process and evolutionary rules of *R. pesudosolanacearum*. In the future, further detection of pathogenicity and genomic changes of RSSC in the same host, analysis of the environmental factors that drive RSSC to mutate and collection of key substances (genes, proteins, etc.) that regulate genomic changes may provide a unique target for the development of new strategies to inhibit RSSC from changing genetic materials.

## Methods

### Plants and strains information and growth conditions

*R. pseudosolanacearum* strains RS639 (sequevar 54), RS642 (sequevar 17), RS647 (sequevar 14) and RS650 (sequevar 13) were collected from sunflower bacterial wilt in the same field at the same time (Collection location: Dongguan city, Guangdong Province, China; collector: Xiaoman She; collection date: 2020–05-18) [20]. Sunflowers were planted as a single crop in this farmland in the first year. The previous crops were some ornamental flowers, and the planting area was about 6.67 hectares. *R. pseudosolanacearum* strains stored in a 20 ℃ storage incubator were revived by incubating on a TZC medium (1 g hydrolyzed casein, 5 g glucose, 10 g peptone, 0.5 g 2,3,5-triphenyltetrazolium chloride, 15 g agar, dissolved in 1 L water, pH 7.2) at 30 ℃ for 48 h. The irregular, round, fluid and white colonies with pink centers were further inoculated into conical flasks containing CPG liquid medium grown overnight at 30 ℃ with shaking at 160 rpm, followed by centrifugation at 10000 rpm for 15 min to collect cells. The prepared strains were used for subsequent experiments. Tomato (*Solanum tuberosum* cv. Dongqie, *S. tuberosum* cv. Xinxing 101), eggplant (*S. melongena* cv. Qingqie, *S. melongena* cv. Nongfu No.2, *S. melongena* cv. Bailong), pepper (*Capsicum annuum* cv. Huifeng No.2, *C. annuum* cv. Yuehong No.3, *C. annuum* cv. Yueshu No.2), tobacco (*Nicotiana tabacum* cv. Dahuangyan) and *Rhizoma kaempferiae* plants used in this study were commercially available. Plants seeds were sowed in soil matrix and grown under the conditions, 18 h light/6 h dark at 28 ~ 36 ℃ and humidity of 62% ~ 85%.

### Plant inoculation and pathogenicity assays

Pathogenicity assays were performed on four- to six-leaf stage plants of tomato, pepper, eggplant, tobacco and *R. kaempferiae*. Fifteen plants of each host were inoculated with four strains. Four hosts (tomato, pepper, eggplant and tobacco) were inoculated by injuring the roots and soaking them in a bacterial suspension (1 × 10^8^ cfu/ml) for 20 min. The roots of 15 plants of each host were also injured and soaked in fluid nutrient medium as negative controls. Fifteen *R. kaempferiae* plants were inoculated by injection with 200 μl of a bacterial suspension (1 × 10^8^ cfu/ml) in the stem bases. Another fifteen *R. kaempferiae* plants were injected with 200 μL of liquid nutrient medium as negative controls. Disease incidence (DI) was monitored every 7 days for 35 days. Plants with wilted leaves were recorded as diseased plants. The disease incidence was calculated as DI (%) = 100 × number of disease plants /15 inoculated plants. This experiment was repeated three times.

### Genome sequencing and assembly

Genomic DNA of four strains was extracted using HiPure Bacterial DNA Kits (Magen, Guangzhou, China) according to the manufacturer’s instructions. The DNA quality was detected using Qubit (Thermo Fisher Scientific, Waltham, MA) and Nanodrop (Thermo Fisher Scientific, Waltham, MA) accordingly. Genome sequencing was performed using a combination of PacBio and Illumina technologies. For PacBio sequencing, qualified genomic DNA was fragmented with G-tubes (Covaris, Woburn, MA, USA) and end-repaired to prepare SMRTbell DNA template libraries (with fragment size of > 10 Kb selected by blue pippin system) according to the manufacturer’s specification (PacBio, Menlo Park, CA). Library quality was detected by Qubit® 2.0 Flurometer (Life Technologies, CA, USA) and average fragment size was estimated on a Bioanalyzer 2100 (Agilent, Santa Clara, CA). SMRT sequencing was performed on the Pacific Biosciences Sequel (PacBio, Menlo Park, CA) according to standard protocols. For Illumina sequencing, genomic DNA was firstly sonicated randomly, and then end-repaired, A-tailed, and adaptor ligated using NEBNext® ΜLtra™ DNA Library Prep Kit for Illumina (NEB, USA) according to the preparation protocol. DNA fragments with length of 300–400 bp were enriched by PCR. At last, PCR products were purified using AMPure XP system (Beckman Coulter, Brea, CA, USA) and libraries were analysed for size distribution by 2100 Bioanalyzer (Agilent, Santa Clara, CA) and quantified using real-time PCR. Genome sequencing was performed on the Illumina Novaseq 6000 sequencer using the pair-end technology (PE 150). Continuous long reads were attained from SMRT sequencing runs and were used for de novo assembly using Falcon (version 0.3.0) [50].

### Genome component prediction

The ORFs (Open reading frames) were predicted using National Center for Biotechnology Information (NCBI) prokaryotic genome annotation pipeline [51]. Noncoding RNAs such as rRNAs prediction was carried out using rRNAmmer (version 1.2) [52] and tRNAs were identified by tRNAscan (version 1.3.1) [53], sRNAs were identified by cmscan (version 1.1.2) [54].. Prophage were identified using PHAST (version 2.0) [55]. Gene Islands were predicted using IslandPath-DIMOB (version 1.0.0) [56].

### Function annotations

The genes were annotated by aligning with the deposited ones in diverse protein databases including NCBI non-redundant protein sequence (Nr) database, UniProt/Swiss-Prot, Kyoto Encyclopedia of Genes and Genomes, Gene Ontology, Cluster of Orthologous Groups of proteins Protein family annotation was applied with Pfam_Scan (version 1.6) basing on Pfam database (version 32.0) [57]. Additional annotation was carried out basing on the following databases: PHI, VFDB. Type III effectors were identified and annotated by the Ralsto T3E database [29], and verified and supplemented through the NCBI Nr database.

### Comparative genomic analysis

The genome sequences of GMI1000, CQPS-1, EP1, Po82, CMR15 and PSI07 was downloaded from NCBI database. Orthologous Average Nucleotide Identity Tool (OAT) was used to measure the overall genome sequences similarity [58]. Genomic alignment between RS639, RS642, RS647, RS650 and the reference genome of GMI1000 were performed using the MUMmer (Version 3.1) [59], and the large-scale co-linearity between the genomes was determined. Gene family were analyzed by bidirectional best-hit standard with 80% of the shortest protein sequences have 40% amino acid similarity. The all amino acid sequences were compared by diamond (Version 2.0.7) [60], and similarity clustering was carried out by OrthoMCL (Version 1.4) [61]. The genetic neighborhood visualization and comparison of gene cluters were anlalyzed by Clinker (https://cagecat.bioinformatics.nl/) [62].

### Supplementary Information

**Supplementary file 1. **

## Data Availability

The complete genomes generated during the current study were deposited in NCBI database: RS639 (Accession number: CP084738-CP084739), RS642 (Accession number: CP086018-CP086019), RS647 (Accession number: CP086265-CP086266) and RS650 (Accession number: CP086104-CP086105).

## Abbreviations

RSSC: *Ralstonia solanacearum* Species complex
ANI: Average nucleotide identify
Sequevar: RSSC are further sub-classified based on the sequence variation
dpi: Days post inoculation
DI: Disease incidence
SMRT: Single Molecule Real-Time
CDS: Complete coding sequence
COG: Clusters of Orthologous Groups
OAT: Orthologous Average Nucleotide Identity Tool
PHI: Pathogen Host Interactions
VFDB: Virulence Factors of Pathogenic Bacteria
GI: Genomic island
T2SS: Type II secretion systems
T3SS: Type III secretion system
T4SS: Type IV secretion system
T6SS: Type VI secretion system
T3Es: Type III secreted effectors
EPS: Exopolysaccharide
NCBI: National Center for Biotechnology Information
